# Multilayered interplay between the cGAS-STING pathway and autophagy

**DOI:** 10.1080/27694127.2026.2728537

**Published:** 2026-09-14

**Authors:** Ze-Bo Huang, Xiang-Zheng Gao, Jin-Yi Lin, Du Feng, Guang Lu

**Affiliations:** a Zhongshan School of Medicine, Sun Yat-Sen University, Guangzhou, China; b Faculty of Health Sciences, Ministry of Education Frontiers Science Center for Precision Oncology, University of Macau, Macau, China; c State Key Laboratory of Respiratory Disease, Guangzhou Municipal and Guangdong Provincial Key Laboratory of Protein Modification and Degradation, School of Basic Medical Sciences, Guangzhou Medical University, Guangzhou, China

**Keywords:** cGAS-STING pathway, innate immunity, lysosome, mitophagy, mtDNA, non-canonical autophagy

## Abstract

The cGAS-STING pathway detects cytosolic dsDNA to initiate innate immune responses, serving as a critical surveillance system against infection and cellular stress. Autophagy is an evolutionarily conserved catabolic process that maintains homeostasis by degrading cytoplasmic components. Although these two systems operate through distinct mechanisms, recent studies have uncovered a complex bidirectional regulatory network that intimately links them. On the one hand, STING has an evolutionarily conserved capacity to induce TBK1-independent noncanonical autophagy, and recent studies further link this activity to TFEB-dependent lysosome biogenesis, expanding its functional repertoire beyond classical interferon induction. On the other hand, autophagy restricts cGAS-STING signaling by clearing cytosolic DNA and selectively degrading pathway components, thereby preventing excessive inflammation. Furthermore, mitophagy curtails the release of mitochondrial DNA, a potent cGAS agonist, and recent studies further implicate lysosomes as active signaling platforms associated with mtDNA release, LRRK2 activation, and STING-dependent NF-κB responses. In this review, we discuss recent advances in understanding how the cGAS-STING pathway and autophagy mutually regulate each other at the cellular level, focusing on the molecular mechanisms of this interplay, both canonical and noncanonical, and highlighting how their crosstalk shapes cellular homeostasis and stress adaptation.

## Introduction

Innate immunity serves as the first line of host defense against pathogen invasion. The innate immune system employs a diverse array of pattern recognition receptors (PRRs) distributed both extracellularly and intracellularly to recognize pathogen-associated molecular patterns (PAMPs) or damage-associated molecular patterns (DAMPs), thereby initiating innate immune responses [1]. A classic example is TLR4 (toll like receptor 4) on the cell surface, which recognizes lipopolysaccharide (LPS) derived from Gram-negative bacteria, leading to NF-κB (nuclear factor kappa B) pathway activation [2]. Inside the cell, there are also receptors that sense viral RNA or DNA. Among these, DNA sensors function as essential components of the innate immune system, not only recognizing and eliminating pathogenic DNA but also sensing endogenous cellular DNA (such as DNA originating from the nucleus or mitochondria) [3]. The first DNA sensor identified was TLR9 (toll like receptor 9) in 2000. Since then, several cytosolic DNA sensors have been discovered, including ZBP1/DAI (Z-DNA binding protein 1), AIM2 (absent in melanoma 2), IFI16 (interferon gamma inducible protein 16), and cGAS (cyclic GMP-AMP synthase) [4]. Extensive genetic and pathological evidence has revealed cGAS as the predominant cytosolic DNA sensor, which recruits the endoplasmic reticulum (ER)-resident protein STING (stimulator of interferon genes) to induce expression of type I interferons (IFNs) and interferon-stimulated genes (ISGs), thereby initiating the canonical cGAS-STING signaling pathway [5,6].

Autophagy is an evolutionarily conserved cellular process and its core machinery is preserved from yeast to mammals. The term “autophagy” derives from the Greek words meaning “self-eating.” It was first introduced by Christian de Duve in 1963 to describe the cellular process by which cytoplasmic components are delivered to lysosomes for degradation. Based on the mode of cargo delivery to lysosomes, autophagy is primarily categorized into macroautophagy, microautophagy, and chaperone-mediated autophagy (also known as CMA). Macroautophagy (hereafter autophagy) stands as the predominant and most intensively studied autophagic pathway, defined by its hallmark feature: the biogenesis of double-membrane autophagosomes that sequester cytoplasmic materials and deliver them to lysosomes for degradation [7]. Autophagy can also be classified according to cargo selectivity. Nonselective autophagy is typically induced under stress conditions such as nutrient starvation, whereas selective autophagy generally relies on cargo receptors that recognize specific substrates through ubiquitin-dependent or ubiquitin-independent mechanisms [8]. Additionally, receptor-independent mechanisms have also been reported. For example, mitochondrial lipids such as cardiolipin and ceramide can contribute to the selective removal of damaged mitochondria [9]. This selective process gives rise to distinct subtypes such as mitophagy (selective autophagy of mitochondria), and ER-phagy (selective autophagy of ER), which play critical roles in organelle quality control and the fine-tuning of intracellular homeostasis [10]. In the 1990s, Yoshinori Ohsumi conducted pioneering genetic screens in yeast, identifying multiple autophagy-related (ATG) genes and elucidating their functions, thereby establishing the molecular foundation for autophagy research [11]. These discoveries have further demonstrated that precise regulation of the autophagy pathway is essential for cell survival. Conversely, its dysfunction is intimately associated with a broad spectrum of major human pathologies, including neurodegenerative diseases, infections, cancer, and cardiovascular disorders [12].

While the cGAS-STING pathway and autophagy execute distinct biological functions, they both are evolutionarily conserved stress sensors that detect aberrant intracellular signals and initiate protective responses to maintain cellular homeostasis. Recent studies have revealed a complex bidirectional regulatory network between these two ancient homeostatic pathways, with the multilayered nature of their interplay gradually coming into focus. Early studies have established that autophagy serves as an important negative regulator of cGAS-STING signaling. By removal of cytosolic nucleic acids and by selectively targeting the core components of this pathway for autophagic degradation, autophagy prevents aberrant hyperactivation of this pathway and hence its consequent detrimental inflammation [13]. Conversely, a series of discoveries has uncovered that the cGAS-STING pathway itself possesses the capacity to induce non-canonical autophagy, and recent studies further demonstrate that STING regulates lysosome biogenesis via mechanisms independent of TBK1 (TANK-binding kinase 1) in cells with viral infection [14–16]. These findings support an evolutionarily conserved autophagic function of STING that may predate its canonical immune signaling. More recent studies have extended this model by linking STING activation to TFEB-dependent lysosome biogenesis. Thus, the functional repertoire of cGAS-STING extends beyond classical interferon induction to encompass direct regulation of autophagic membrane dynamics, which offers a new conceptual framework for understanding its broader biological significance.

In this review, we systematically synthesize recent advances in the bidirectional crosstalk between the cGAS-STING pathway and autophagy. We focus on two central questions: how cGAS-STING acts as an upstream signal to initiate autophagy, and how autophagy regulates cGAS-STING activity. We further examine the molecular mechanisms underlying this interactive network and its functional implications in cellular homeostasis. Finally, we conclude by highlighting key questions that remain unanswered and outlining promising directions for future investigation. A deeper understanding of the interplay between the cGAS-STING pathway and autophagy will not only illuminate fundamental principles governing cellular stress responses but also provide a theoretical foundation for therapeutic strategies targeting these pathways in human disease.

## The cGAS-STING signaling pathway

### Canonical signaling of the cGAS-STING pathway

**Figure 1.**
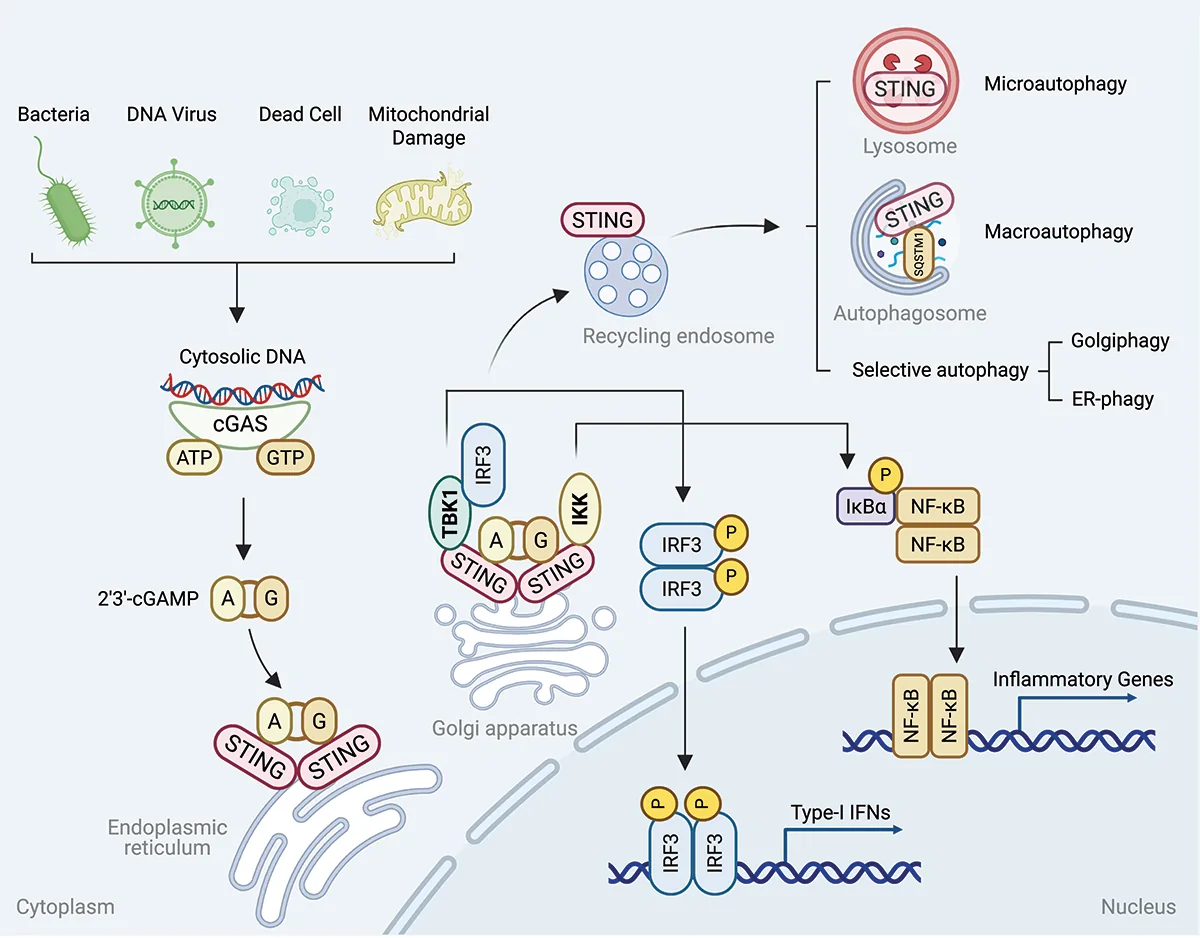
The cGAS-STING signaling pathway and its regulation by autophagy. Cytosolic DNA from diverse sources, including bacteria, DNA viruses, dead cells, and damaged mitochondria, triggers activation of cGAS, which synthesizes 2′3′-cGAMP using ATP and GTP. cGAMP then binds and activates STING, promoting its translocation from the endoplasmic reticulum to the Golgi apparatus. At the Golgi, activated STING oligomerizes and recruits the downstream kinases TBK1 and IKK. TBK1 mediates phosphorylation and dimerization of IRF3, driving its nuclear translocation and subsequent transcription of type I IFN genes. In parallel, IKK activation leads to IκBα phosphorylation and degradation, releasing NF-κB to translocate into the nucleus and induce expression of the inflammatory genes. To prevent excessive signaling, STING traffics from the Golgi to recycling endosomes (REs) and then to lysosomes for degradation, a canonical signal-termination route. This degradation is mediated by multiple autophagic pathways, including microautophagy, macroautophagy (via SQSTM1), Golgiphagy, and ER-phagy, which collectively provide negative feedback to terminate STING signaling. The diagram is generated using BioRender (https://BioRender.com).

Canonical signaling of the cGAS-STING pathway is initiated upon detection of cytosolic double-stranded DNA (dsDNA) by cGAS (Figure 1). Under normal conditions, cGAS resides in both the cytosol and the nucleus. Within the nucleus, cGAS is tethered to nucleosomes which occupy its DNA-binding site, thereby suppressing its enzymatic activity [17–21]. Moreover, the CRL5 (cullin-RING ubiquitin ligase 5)-SPSB3 (splA/ryanodine receptor domain and SOCS box containing 3) ubiquitin ligase complex has been revealed to control the abundance of cGAS in the nucleus by targeting it for proteasomal degradation to inhibit its enzymatic activity [22]. In the cytosol, the nuclear envelope serves as a physical separation between nuclear DNA and cytoplasmic cGAS to prevent aberrant innate immunity. It has been revealed that during the transient opening of the nuclear envelope such as at the end of mitosis, the chromatin architectural protein BANF1/BAF (barrier to autointegration nuclear assembly factor 1) restricts cGAS not only by competing for DNA but also by displacing cGAS already bound to chromatin during transient nuclear-envelope opening [23]. These mechanisms collectively restrain the activation of cGAS by genomic DNA. The presence of pathogen DNA in the cytosol or self-DNA released from the nucleus or mitochondria is thus the major cause for activation of the canonical cGAS-STING signaling pathway.

Upon binding to cytosolic dsDNA, cGAS undergoes a conformational change, resulting in the synthesis of the second messenger 2’3’-cyclic GMP-AMP (2’3’-cGAMP) from ATP (adenosine triphosphate) and GTP (guanosine triphosphate). Notably, this binding of cGAS to cytosolic dsDNA drives the formation of liquid-like droplets through liquid-liquid phase separation (LLPS), which dramatically lowers the concentration threshold for cGAS activation, enabling ultrasensitive detection of cytosolic DNA and a burst of cGAMP production [24]. cGAMP then binds to STING dimer residing at the ER to promote its oligomerization, which promotes the translocation of STING from the ER to the Golgi apparatus via the ER-Golgi intermediate compartment (ERGIC). At the Golgi, STING recruits TBK1 through its C-terminal tail. TBK1 is subsequently activated by trans-autophosphorylation and in turn phosphorylates STING at Ser366 within its C-terminal tail [5]. This phosphorylation event creates a docking site for IRF3 (interferon regulatory factor 3), allowing TBK1 to phosphorylate IRF3. Phosphorylated IRF3 then dimerizes and translocates to the nucleus, where it drives the expression of type I interferons and downstream interferon-stimulated genes (ISGs) [25].

In parallel, activated TBK1, together with its homolog IKKε (inhibitor of NF-κB kinase subunit epsilon) in certain immune cells, phosphorylates and activates the IKK complex comprising IKKα (inhibitor of NF-κB kinase subunit alpha), IKKβ (inhibitor of NF-κB kinase subunit beta), and NEMO/IKKγ (inhibitor of NF-κB kinase subunit gamma) [26,27]. The activated IKK complex (primarily through IKKβ) then phosphorylates IκBα (NF-κB inhibitor alpha), targeting it for ubiquitin-dependent degradation and thereby releasing NF-κB (typically the p50/p65 heterodimer) to translocate into the nucleus and induce pro-inflammatory cytokine gene expression [28]. Notably, NEMO-IKKβ signaling can also be activated independently of cGAS-STING. In response to mitochondrial damage, PRKN (parkin RBR E3 ubiquitin protein ligase)-dependent ubiquitination recruits NEMO and activated IKKβ to mitochondria, which provides an alternative route to NF-κB activation [29].

### Phosphoinositides modulate STING activation

Emerging evidence suggests that, beyond cGAMP binding, specific phosphoinositides could facilitate STING trafficking and higher-order oligomerization. Two recent studies have identified phosphatidylinositol-3,5-bisphosphate (PtdIns(3,5)P_2_) as an endogenous ligand of STING [30,31]. STING constitutively associates with the PIKFYVE (phosphoinositide kinase, FYVE-type zinc finger containing) complex, which comprises PIKFYVE, VAC14 (VAC14 component of PIKFYVE complex), and FIG4 (FIG4 phosphoinositide 5-phosphatase), and the lipid kinase PIKFYVE mediates the synthesis of PtdIns(3,5)P_2_ from phosphatidylinositol-3-phosphate (PtdIns3P). Although PtdIns(3,5)P_2_ is poorly enriched at the ER, its binding to STING has been reported to promote ER exit and further oligomerization on post-Golgi/endolysosomal compartments where PtdIns(3,5)P_2_ is enriched [32]. Cryo-electron microscopy (cryo-EM) structures reveal that phosphatidylinositol phosphates (PtdInsPs), together with cholesterol, bind at the interface between STING dimers, directly facilitating higher-order STING oligomerization [30]. These findings provide a mechanistic explanation for the long-standing observation that STING must transit from the ER to the Golgi and post-Golgi vesicles to achieve full activation and highlight how intracellular trafficking coordinates with lipid metabolism to dictate STING signaling output.

Apart from PtdIns(3,5)P_2_, phosphatidylinositol-4,5-bisphosphate (PtdIns(4,5)P_2_) could also bind STING to strongly promote its activation. Several lines of evidence suggest that PtdIns(3,5)P_2_ plays a more specific role in cells. STING is constitutively associated with the PIKFYVE complex, which generates PtdIns(3,5)P_2_ primarily on late endosomes and lysosomes [33], organelles closely linked to STING activity. By contrast, PtdIns(4,5)P_2_ is enriched at the plasma membrane and is present at much lower levels in the Golgi and endosomes [33,34]. Moreover, STING mutants that impair PtdIns(3,5)P_2_ binding exhibit defects in ER-to-Golgi trafficking [30]. Phosphatidylinositol 4-phosphate (PI4P) has also been implicated in STING activation at the Golgi [35], but its effect on STING oligomerization is considerably weaker compared with PtdIns(3,5)P_2_ or PtdIns(4,5)P_2_. Specifically, cGAMP combined with PI4P induces shorter and less abundant STING oligomers than those formed in the presence of PtdIns(3,5)P_2_ or PtdIns(4,5)P_2_ [30].

These observations raise the possibility of a graded activation model, in which different phosphoinositides may fine-tune STING signaling according to cellular context. PtdIns(3,5)P_2_ and PtdIns(4,5)P_2_, by promoting higher-order oligomerization, may support stronger STING activation. In contrast, PI4P, which induces only shorter oligomers, may produce a weaker signaling response. However, the general applicability of this graded activation model remains to be validated across broader cellular and physiological contexts. It will also be important to determine how the contribution of each phosphoinositide varies across membrane compartments, cell types, and activating conditions.

### DNase-mediated cytosolic DNA clearance restrains the cGAS-STING signaling

Timely clearance of cytosolic DNA is essential to prevent aberrant activation of the cGAS-STING pathway. Among the regulators, TREX1 (three prime repair exonuclease 1) has emerged as an essential 3′→5′ DNA exonuclease to limit aberrant STING signaling via degradation of cytosolic DNA [36]. Genetic mutations of TREX1 are implicated in various autoimmune diseases such as Aicardi-Goutières syndrome (AGS) and systemic lupus erythematosus (SLE) [37–39]. In animal models, whole-body deletion of Trex1 leads to lethal autoimmunity characterized by systemic inflammation, tissue damage, and autoantibody production, which is alleviated upon cGAS ablation [40,41]. Similarly, deficiency in the lysosomal DNase II also triggers cGAS-dependent inflammation, further establishing cGAS as the key effector downstream of failed self-DNA clearance [40]. Beyond these genetic links, downregulation of cytoplasmic DNases, including TREX1 and DNase II contribute to activation of the cGAS-STING pathway, and induction of the senescence-associated secretory phenotype (SASP) during cellular senescence [42].

TREX1 is anchored to the ER and the outer nuclear membrane. Upon rupture of the micronuclear envelope, this ER-anchored TREX1 gains access to the micronucleus and degrades the exposed DNA, thereby restricting cGAS activation at these sites [43]. Importantly, SLE-associated TREX1 mutations disrupt its ER association, leading to impaired micronuclear DNA degradation and aberrant activation of the cGAS-STING signaling [43]. These findings identify defective TREX1-mediated clearance of micronuclear DNA as an important mechanism that contributes to TREX1-associated autoimmunity through cGAS-STING activation. In parallel, cGAS itself has evolved a counter-mechanism to resist TREX1-mediated clearance of cytosolic DNA. Upon binding DNA, cGAS undergoes LLPS which provides a selective environment to suppress TREX1 exonuclease activity and hence to block DNA degradation [44]. A recent study reports that TREX1 employs a modular dsDNA-recognition architecture, including a highly conserved arginine residue (R128) and an auxiliary DNA-binding surface, to engage dsDNA and compete with cGAS in phase-separated condensates, thereby setting a tunable activation threshold for the cGAS-STING pathway [45]. These findings suggest that TREX1 may have a more active role in dsDNA recognition than previously appreciated, beyond its established exonuclease activity.

These studies indicate that TREX1 and cGAS compete for access to cytosolic DNA. This competition is influenced not only by their DNA-binding properties, but also by cGAS-DNA condensate formation and the localization of TREX1. Whether the same competition occurs when cGAS encounters DNA from other sources, such as mitochondrial or microbial DNA, and what determines whether these DNA molecules are degraded by TREX1 or retained for immune sensing, remain important questions.

## Mechanisms and regulations of autophagy

### The core autophagy machinery

**Figure 2.**
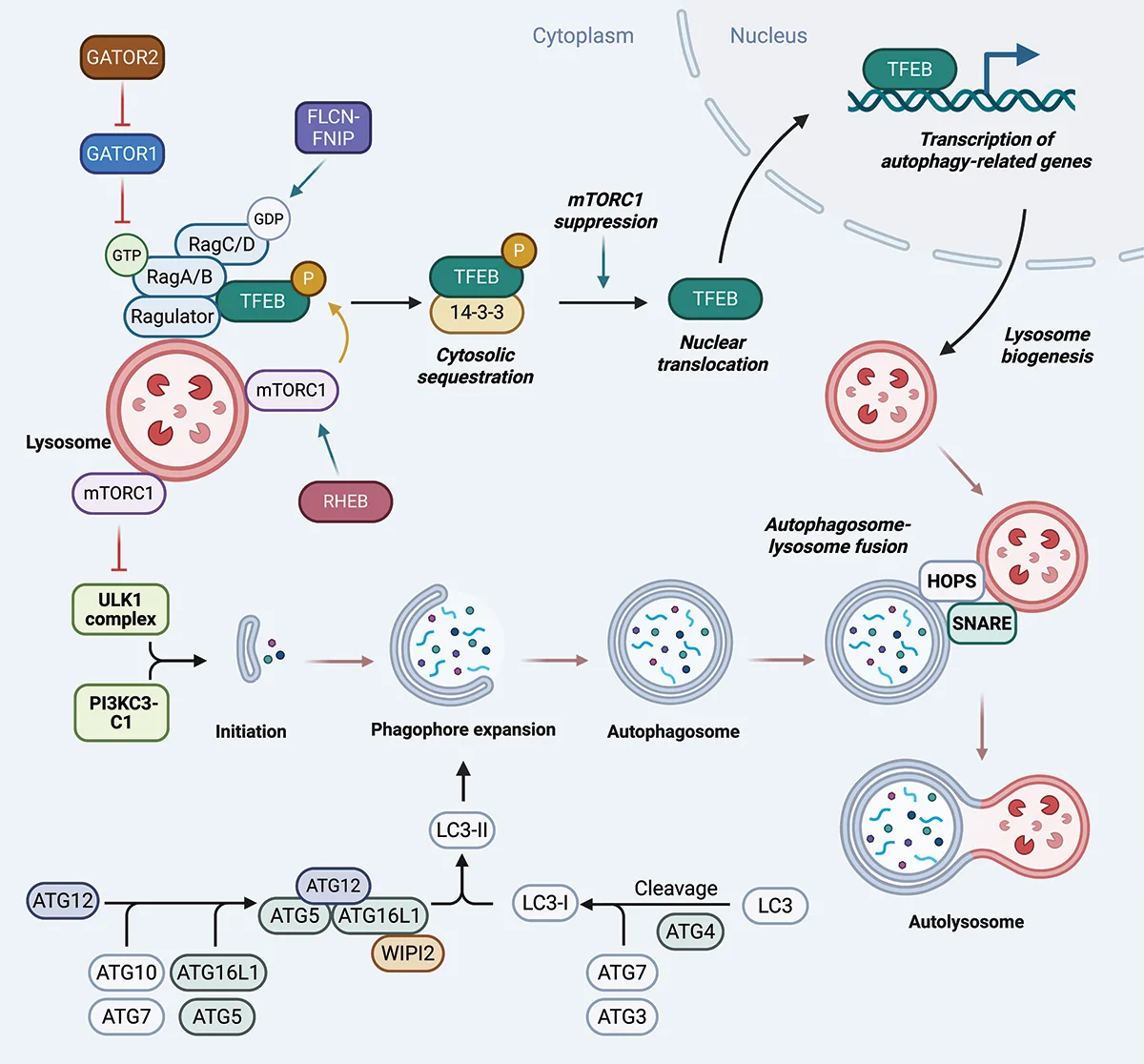
The mTORC1-TFEB regulatory axis and the core macroautophagy pathway. At the lysosomal surface, mTORC1 is activated by the coordinated actions of Rag GTPases (regulated by the upstream GATOR2-GATOR1 cascade and the FLCN-FNIP complex) and GTP-bound RHEB. Under nutrient-rich conditions, activated mTORC1 phosphorylates TFEB, promoting its binding to 14–3-3 proteins and retention in the cytosol. Upon nutrient starvation or mTORC1 suppression, TFEB is dephosphorylated, translocates to the nucleus, and transcriptionally activates autophagy- and lysosome-related genes to drive lysosome biogenesis. Meanwhile, mTORC1 inhibition relieves its repression of the ULK1 complex, which then initiates autophagosome formation with the coordination of PIK3C3-C1. The nascent phagophore expands and matures into a closed autophagosome, which subsequently fuses with lysosomes via HOPS complex- and SNARE-dependent mechanisms to form an autolysosome, where cargo is degraded. LC3 lipidation is critical for the phagophore expansion and maturation, which is mediated by the two ubiquitin-like conjugation systems. Specifically, LC3 is first processed by ATG4 to generate cytosolic LC3-I, then conjugated to phosphatidylethanolamine (PE) to produce membrane-associated LC3-II, a step requiring ATG7, ATG3, and the ATG12–ATG5-ATG16L1 complex (assembled via ATG7, ATG10, and ATG16L1). Arrows denote positive regulation or stepwise progression, while T-bars indicate inhibitory interactions. The diagram is generated using BioRender (https://BioRender.com).

Autophagy encompasses through a series of distinct stages, including initiation, phagophore nucleation and expansion, autophagosome closure, and finally fusion with lysosomes. This process is orchestrated by a set of ATG proteins, of which more than 40 have been identified to date. These ATG proteins assemble into several functional complexes that coordinate the sequential steps of autophagosome formation (Figure 2). Given the availability of comprehensive reviews that extensively cover the molecular details of autophagy, we will not recapitulate the entire pathway here (for detailed mechanisms, see [12]). Instead, we focus on the core components and concepts that are most relevant to the crosstalk with the cGAS-STING pathway in this review.

Autophagosome formation is initiated by activation of the ULK1 (unc-51 like autophagy activating kinase 1) complex. This complex comprises ULK1, ATG13, RB1CC1/FIP200 (RB1 inducible coiled-coil 1), and ATG101. Under nutrient-rich conditions, this complex is kept inactive by mechanistic target of rapamycin complex 1 (mTORC1)-mediated phosphorylation. Upon autophagy induction, ULK1 undergoes autophosphorylation and phosphorylates its associated subunits, triggering its translocation to the phagophore assembly site (PAS) at the ER. At the PAS, the ULK1 complex activates the class III phosphatidylinositol 3-kinase complex I (PI3KC3-C1) that consists of PIK3C3/VPS34 (phosphatidylinositol 3-kinase catalytic subunit type 3), PIK3R4/VPS15 (phosphoinositide-3-kinase regulatory subunit 4), BECN1 (Beclin 1), and ATG14, resulting in the production of PtdIns3P [46]. PtdIns3P serves as a platform for recruiting PtdIns3P-binding effectors, primarily members of the WIPI (WD-repeat protein interacting with phosphoinositides) family, which promote membrane expansion. The WIPI family comprises four members (WIPI1, WIPI2, WIPI3 and WIPI4), each with distinct roles in autophagosome formation [47].

WIPI2 (WD repeat domain, phosphoinositide interacting 2) is a major regulator of MAP1LC3/LC3 (microtubule associated protein 1 light chain 3) lipidation. It directly binds PtdIns3P at the PAS and recruits the ATG12–ATG5-ATG16L1 complex through interaction with ATG16L1. This complex functions as an E3-like ligase that catalyzes the conjugation of ATG8 family proteins to phosphatidylethanolamine (PE) [48]. The mammalian ATG8 family includes LC3A, LC3B, LC3C, GABARAP (GABA type A receptor-associated protein), GABARAPL1 (GABA type A receptor associated protein like 1), and GABARAPL2 (GABA type A receptor associated protein like 2). Their lipidation proceeds through two ubiquitin-like conjugation systems (Figure 2). In the first, ATG12 is conjugated to ATG5 with the help of ATG7 and ATG10. The resulting ATG12–ATG5 conjugate then binds ATG16L1. In the second, ATG8 proteins are first cleaved by ATG4 to expose a C-terminal glycine, then activated by ATG7, transferred to ATG3, and finally conjugated to PE. Lipidated ATG8 proteins anchor to the expanding phagophore membrane, promoting membrane curvature and serving as docking sites for selective autophagy receptors such as SQSTM1/p62 (sequestosome 1), which recognizes ubiquitinated cargo [49].

ATG9 is the only transmembrane ATG protein and plays a critical role in membrane delivery during autophagosome formation. Under basal conditions, endogenous ATG9 localizes to both the trans-Golgi network (TGN) and late endosomes, and it constitutively cycles between these compartments [50]. Upon autophagy induction, ATG9 redistributes from the juxta-nuclear TGN to peripheral endosomal compartments dependent on ULK1-mediated phosphorylation, a process essential for supplying membrane to the expanding phagophore [50–52]. ATG9 then collaborates with ATG2 and WIPI4 to establish ER-phagophore contact sites. At these contact sites, ATG2 mediates lipid transfer from the ER to the outer leaflet of the phagophore, while ATG9 facilitates lipid flip-flop between the two leaflets. This coordinated action supplies the membrane necessary for phagophore expansion. WIPI2 also participates in tethering the ER to the phagophore through its interaction with the ULK1 complex and ER-resident vesicle-associated membrane protein (VAMP)-associated proteins (VAPs) [53]. This ER-phagophore contact is thought to stabilize the assembly site and may facilitate the coordination of membrane supply with the expanding phagophore.

The final step of autophagy involves the fusion of mature autophagosomes with lysosomes. This process that is tightly regulated by the homotypic fusion and protein sorting (HOPS) complex and the SNARE (soluble N-ethylmaleimide-sensitive factor attachment protein receptor) proteins (Figure 2). The HOPS complex is a hexameric tethering complex (composed of VPS11, VPS16, VPS18, VPS33A, VPS39, and VPS41) that binds to both autophagosomes and lysosomes, drawing the two organelles into close apposition and setting the stage for SNARE-mediated membrane fusion [54]. Fusion is mediated by the formation of trans-SNARE complexes that bridge the two membranes. On the autophagosome, STX17 (syntaxin 17) assembles with the Qbc-SNARE SNAP29 (synaptosomal-associated protein 29) and the lysosomal R-SNARE VAMP8 to form the canonical STX17-SNAP29-VAMP8 complex. The HOPS tethering complex binds directly to STX17, bringing the two organelles into close apposition and setting the stage for SNARE assembly and subsequent membrane fusion [55]. In parallel, alternative SNARE complexes such as STX17-SNAP29-VAMP7 can also function in fusion. Furthermore, YKT6 (YKT6 vesicular SNARE protein) plays a non-canonical priming role by forming a YKT6-STX17-SNAP29 complex that facilitates HOPS recruitment and is subsequently replaced by VAMP8 to generate the fusogenic complex [56,57].

### The mTORC1-TFEB axis in regulation of autophagy

Autophagy is tightly controlled by upstream signaling pathways that integrate cellular nutrient and energy status. The mTORC1 serves as a central metabolic checkpoint that suppresses autophagy under nutrient-rich conditions. mTORC1 localizes to the lysosomal surface, where its activation is regulated by amino acid availability through a signaling cascade that converges on the Ras-related GTP binding (RAG) GTPases [58]. The RAGs function as a heterodimeric switch: when loaded with GTP-bound RAG A/B and GDP-bound RAG C/D, they recruit mTORC1 to the lysosome; the opposite nucleotide configuration renders them inactive. The guanine nucleotide state of the RAGs is controlled by two distinct GTPase-activating proteins (GAPs), with GATOR1 (Gap Activity Toward Rags 1) acting on RAG A/B and FLCN (folliculin)-FNIP (folliculin interacting protein) acting on RAG C/D (Figure 2). Under amino acid-rich conditions, GATOR2 (GTPase activating proteins toward Rags subcomplex 2) antagonizes GATOR1, relieving its GAP activity and allowing RAG A/B to accumulate in the GTP-bound form [59,60]. In parallel, the FLCN-FNIP complex functions as a GAP for RAG C/D, promoting their conversion to the GDP-bound state. Consequently, the active RAG heterodimer (comprising RAG A/B-GTP and RAG C/D-GDP) binds and recruits mTORC1 to the lysosomal surface, where the lysosome-resident small GTPase RHEB (Ras homolog, mTORC1 binding) further activates mTORC1 kinase activity [61]. Upon amino acid deprivation, GATOR2 is inhibited, enabling GATOR1 to hydrolyze GTP on RAG A/B, converting them to the inactive GDP-bound state [62]. This prevents mTORC1 from associating with the lysosomal membrane, thereby shutting down its downstream signaling.

Autophagy is suppressed by mTORC1 through multiple mechanisms. mTORC1 directly phosphorylates the ULK1 complex, maintaining it in an inactive state [62]. Simultaneously, mTORC1 phosphorylates TFEB (transcription factor EB) to enhance TFEB cytoplasmic retention via interaction with 14–3-3 proteins, thereby inhibiting the transcription activity of TFEB essential for the expression of autophagy and lysosomal genes [63]. Conversely, lysosomal calcium release activates the phosphatase calcineurin, which dephosphorylates TFEB and promotes its nuclear translocation [64]. These regulatory mechanisms ensure that autophagy is precisely tuned to cellular metabolic demands. Notably, certain components of the core autophagy machinery, particularly ATG16L1 and the LC3 lipidation system, have been implicated in non-canonical autophagy that operates independently of the upstream ULK1 and VPS34 complexes. These alternative pathways will be discussed in detail in subsequent sections.

## The cGAS-STING pathway elicits non-canonical autophagy

### Coordination of cGAS-STING signaling and autophagy to restrict microbial infection

Prior to the elucidation of the molecular mechanisms underlying the regulation of autophagy by the cGAS-STING pathway, numerous studies had documented the simultaneous activation of cGAS-STING signaling and autophagy induction, which together contribute to cell-intrinsic defense against both viral and bacterial pathogens. Upon DNA virus infection, cGAS-STING signaling induces autophagy through TRIM23 (tripartite motif containing 23)-mediated TBK1 activation, which in turn phosphorylates the selective autophagy receptor p62 to target viral particles for autophagic degradation and thereby restricts viral replication [65]. In bacterial infection, mycobacterium tuberculosis DNA is sensed by cGAS in infected macrophages, which not only triggers type I interferon production but also induces autophagy that targets bacteria to lysosomes for degradation [66,67]. cGAS knockout or knockdown severely impairs autophagy induction, and cGAS-deficient mice are more susceptible to *M. tuberculosis* infection [67]. Moreover, extracellular bacterial DNA triggers ubiquitination of bacteria via a STING-dependent manner, which enables the autophagy receptors p62 and CALCOCO2/NDP52 (calcium binding and coiled-coil domain 2) to recognize ubiquitinated bacteria for autophagic clearance [68].

The crosstalk between the core components of cGAS-STING and autophagy is bidirectional upon pathogen infections. While microbial DNA activates the cGAS-STING pathway, the direct binding of cGAS to Beclin1 simultaneously inhibits cGAS enzymatic activity and enhances canonical autophagy via releasing RUBCN/Rubicon (rubicon autophagy regulator) from the Beclin1 complex, resulting in autophagic clearing cytosolic microbial DNA [69,70]. Conversely, autophagy serves as a critical brake on the cGAS-STING pathway to maintain immune homeostasis (Figure 1). Following activation, ULK1 phosphorylates STING to promote its autophagic degradation, while TBK1 phosphorylates p62 to enhance recognition of ubiquitinated STING, collectively terminating innate immune signaling [71,72]. Moreover, STING harboring a gain-of-function mutation (R284S, which causes constitutive activation without cGAMP stimulation) could also be targeted for degradation by ULK1-dependent autophagy to prevent uncontrolled inflammation [73]. Upon the late stage of viral infection, SENP3 (SUMO specific peptidase 3) mediates the deSUMOylation of cGAS and STING, resulting in the degradation of STING via the CMA pathway [74]. This shift from SUMOylation-dependent stabilization during the early phase of infection to deSUMOylation-dependent degradation at the late phase may allow the pathway to support an initial antiviral response while preventing prolonged activation. Therefore, autophagy restrains cGAS-STING signaling at different stages of infection. It removes microbes and microbial DNA during the initial response, and later contributes to the degradation of the key cGAS-STING pathway components to terminate the signaling.

### STING activates non-canonical autophagy via TBK1-independent mechanisms

**Figure 3.**
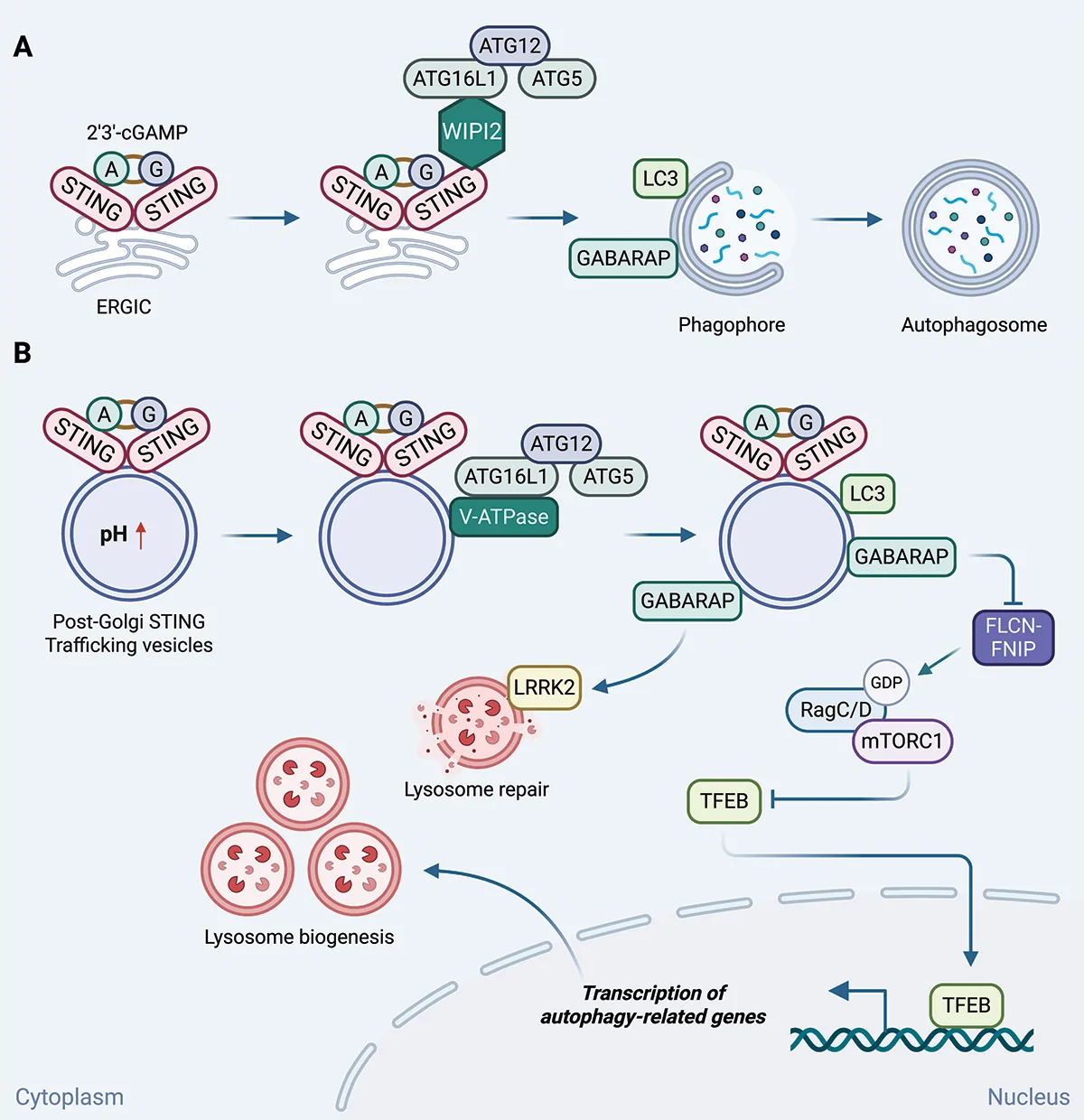
STING-initiated non-canonical autophagy and its roles in lysosomal homeostasis. (**A**) STING triggers non-canonical autophagy at the ERGIC. Upon activation by 2′3′-cGAMP, STING at the ERGIC directly interacts with WIPI2, which in turn recruits the ATG12–ATG5-ATG16L1 complex. This cascade drives LC3 and GABARAP lipidation at the phagophore, ultimately promoting the formation of double-membrane autophagosomes. (**B**) post-Golgi STING trafficking vesicles mediate LC3/GABARAP lipidation and support lysosomal quality control. STING itself functions as a proton channel to control the luminal pH of the single-membrane STING positive vesicle. Maturing post-Golgi STING vesicles with elevated luminal pH trigger the recruitment of V-ATPase. V-ATPase then interacts with ATG16L1 to recruit the ATG12–ATG5-ATG16L1 complex, which promotes lipidation of LC3 and GABARAP on these vesicles. Lipidated GABARAP inhibits the activity of the FLCN-FNIP axis, resulting in suppression of mTORC1 and subsequent nuclear translocation of the MiT/TFE family of transcription factors (including TFEB, TFE3, and MITF). These factors then drive the transcription of autophagy- and lysosome-related genes, thereby promoting lysosome biogenesis. Additionally, lipidated GABARAP facilitates the recruitment of LRRK2 to the lysosomal surface, where LRRK2 activation promotes ESCRT-mediated lysosomal repair. The diagram is generated using BioRender (https://biorender.com).

Whereas earlier studies had documented the concurrent activation of cGAS-STING signaling and autophagy in response to pathogen invasion, two independent studies published in 2019 established a direct link between STING trafficking and autophagy [16,75]. One of the key conceptual advances in the field is the discovery that the cGAS-STING pathway can directly induce a form of non-canonical autophagy that is independent of the canonical ULK1 and VPS34-Beclin1 complexes (Figure 3). Importantly, this function is evolutionarily conserved, as it is present in organisms like the sea anemone *Nematostella vectensis* that lack the TBK1-IRF3 signaling axis, suggesting that this autophagy function of the cGAS-STING pathway predates its role in inducing type I interferons [16]. Mechanistically, upon binding to cGAMP, STING translocates from the ER to the ERGIC in a COP II- and ARF GTPase 1 (ARF1)-dependent manner [16]. STING-containing ERGIC then serves as a membrane source for LC3 lipidation, generating conventional double-membrane autophagosomes, which requires WIPI2 and ATG5 but bypasses the ULK1 and VPS34-Beclin1 complexes [16]. The direct molecular link between STING and WIPI2 has further been elucidated. STING binds WIPI2 through its C terminal domain, and disruption of this interaction impairs LC3 lipidation on STING-containing vesicles and subsequent autophagosome formation (Figure 3A) [76,77]. Of note, STING binds WIPI2 at the same FRRG motif on WIPI2 that is otherwise required for PtdIns3P recognition during canonical autophagy, thereby establishing a competition between STING-induced and PtdIns3P-dependent autophagy [76]. Functionally, these studies indicate that STING-mediated WIPI2-dependent autophagy is important for the clearance of DNA and virus in the cytosol [16,76].

STING activation also triggers a WIPI2-independent form of non-canonical autophagy that drives ATG8 lipidation on single-membrane vesicles derived from Golgi, which relies on the vacuolar ATPase (V-ATPase) and the ATG16L1 WD40 domain (Figure 3B) [78]. Upon STING activation, V0 and V1 subunits of the V-ATPase undergo increased engagement, serving as a signaling platform that recruits ATG16L1 via its K490 residue within the WD40 domain to drive LC3 lipidation on the perinuclear single-membrane vesicles [79]. Moreover, this function is preserved in STING with the deletion of the CTT domain (the C-terminal tail that mediates TBK1/IRF3 recruitment for type I interferon responses) [80]. Recent studies reported that STING itself functions upstream to activate this V-ATPase-dependent pathway through its proton channel activity. In this model, proton efflux increases the luminal pH of STING-positive vesicles, which may promote V-ATPase engagement and activation of the V-ATPase-ATG16L1 axis resulting in LC3 lipidation [81,82]. However, the physiological contribution of STING proton channel activity and its relevance across different cell types and *in vivo* settings remain to be established. Unlike double-membrane autophagosomes that target substrates for degradation, this pathway is thought to mediate cell-autonomous host defense that arose early in evolution, even before the emergence of the interferon pathway. A key piece of evidence supporting this defense function comes from studies of the bacterial effector Salmonella outer protein F (SopF), an ADP-ribosyltransferase secreted by *Salmonella*. SopF directly targets the V-ATPase subunit ATP6V0C (ATPase H+ transporting V0 subunit c), modifying it to disrupt the V-ATPase-ATG16L1 interaction and thereby block STING-induced LC3 lipidation onto single-membrane vesicles [78,79,83]. This specific targeting indicates that bacterial effectors such as SopF counteract STING-mediated noncanonical autophagy during infection, highlighting the importance of this pathway in host defense. Moreover, a recent study reported that STING-induced LC3-positive vesicles can also originate from the TGN and these vesicles participate in the unconventional secretion of pro-inflammatory cytokines such as interleukin 1 beta (IL1B) [84], expanding the functional repertoire of STING-induced non-canonical autophagy beyond host defense.

Together, these findings indicate that STING trafficking can induce two mechanistically distinct forms of non-canonical autophagy. At the ERGIC, STING recruits WIPI2 to promote double-membrane autophagosome formation and cytosolic cargo clearance, whereas on post-Golgi single-membrane vesicles, STING activates the V-ATPase-ATG16L1 pathway involved in host defense and unconventional secretion. Whether these two processes represent successive stages of the same trafficking process or separate pathways remains to be explored.

### STING-dependent activation of TFEB and lysosome biogenesis

Between late 2024 and early 2025, multiple independent groups reported that STING also regulates lysosome biogenesis at the transcriptional level via the activation of the transcription factors TFEB and its paralogs TFE3 (transcription factor binding to IGHM enhancer 3) and MITF (melanocyte-inducing transcription factor) (collectively referred to as the MiT/TFE family) (Figure 3B). Based on these studies, STING activation triggers GABARAP lipidation on single-membrane vesicles via the V-ATPase-ATG16L1 axis. Lipidated GABARAP then binds the FLCN-FNIP complex, which inactivates mTORC1 and relieves TFEB phosphorylation, allowing TFEB to translocate into the nucleus and drive lysosomal gene expression [85–87]. This process is independent of TBK1 and has been proposed to represent an evolutionarily conserved function of STING. Further, STING-mediated TFEB activation was reported to depend on its proton channel activity [86,87]. Although these findings provide important mechanistic insights, their physiological and disease relevance remains incompletely defined. To date, the evidence has been derived predominantly from cultured-cell models of viral infection or stimulation with synthetic nucleic acid agonists, with relatively limited validation in animal models or disease settings.

In addition to driving lysosome biogenesis, recent studies further suggest that STING-induced noncanonical autophagy may contribute to lysosomal quality control and repair. STING-induced non-canonical autophagy activates LRRK2 (leucine-rich repeat kinase 2), a kinase linked to Parkinson’s disease (PD), and promotes the recruitment of the ALIX (ALG-2 interacting protein X)-mediated ESCRT (endosomal sorting complexes required for transport) machinery to alleviate endolysosomal membrane damage [87]. Furthermore, in mouse models of lysosomal storage disorders (LSDs), the STING-TFEB axis not only induces lysosomal gene expression but also mediates lysosomal repair [88]. Together, these findings indicate that STING-induced non-canonical autophagy promotes both lysosome biogenesis and lysosomal repair and supports an emerging link between STING signaling and lysosomal homeostasis.

## Autophagy modulates the cGAS-STING pathway

### Termination of the STING signaling via endolysosomal trafficking and autophagy

The termination of STING signaling is tightly coupled to its intracellular trafficking itinerary. Following activation at the ER and translocation to the Golgi, STING continues to be transported to recycling endosomes (REs) and ultimately to lysosomes for degradation (Figure 1) [89,90]. During this process, K63-linked ubiquitination of STING at residue K288 serves as a key signal that recruits the ESCRT, which then mediates the direct encapsulation of STING-positive vesicles derived from REs into LAMP1-positive lysosomes, a process referred to as ESCRT-dependent microautophagy [90]. This STING ubiquitination is mediated by UBE2N (ubiquitin conjugating enzyme E2 N) and driven by STING oligomerization, which forms a platform to recruit ESCRT at the endosome that blocks the STING signaling via sorting in the lysosome [91]. Thus, STING trafficking from the Golgi to recycling endosomes is not only required for signal transduction but also represents an important route for lysosomal degradation of activated STING and contributes to signal termination. This trafficking-degradation coupling ensures precise spatiotemporal control of STING signaling and is essential for immune homeostasis.

A recent study identified TAX1BP1-directed Golgiphagy as an additional route contributing to STING downregulation. Specifically, STING activation induces fragmentation and swelling of the Golgi apparatus. These fragmented Golgi membranes are then recognized by the autophagy receptor TAX1BP1 (Tax1 binding protein 1) together with p62 and subsequently cleared via Golgiphagy, which provides an additional layer of negative feedback regulation of the STING signaling [92]. In addition to Golgiphagy, a recently reported study showed that STING can also be degraded via ER-phagy. The G protein-coupled receptor ADGRE5/CD97 (adhesion G protein-coupled receptor E5) suppresses STING-mediated type I interferon responses by recruiting the ER-phagy receptor RETREG1/FAM134B (reticulophagy regulator 1) to initiate ER-phagy, which results in the degradation of STING by autophagy following DNA virus infection [93]. Therefore, the route of STING degradation is closely linked to its subcellular location. Endosomal microautophagy removes STING-containing vesicles after Golgi exit, whereas Golgiphagy and ER-phagy clear STING together with the Golgi or ER membranes in which it resides.

### Autophagy receptors-mediated degradation of the key components of the cGAS-STING pathway

Autophagy receptors contribute to the negative regulation of the cGAS-STING pathway by targeting its core components for lysosomal degradation. Following activation, STING is ubiquitinated and recognized by the autophagy receptor p62, which has been phosphorylated by TBK1 to enhance its affinity for ubiquitinated STING, thereby delivering STING to autophagosomes [72]. This process is controlled by SUMOylation. The E3 SUMO ligase TRIM38 (tripartite motif containing 38)-mediated SUMOylation of cGAS and STING protects both from degradation during early viral infection, whereas deSUMOylation by SENP2 (SUMO specific peptidase 2) in the late phase promotes the proteasomal degradation of cGAS and the autophagic degradation of STING via CMA [74]. More recently, SESN1 (sestrin 1) was shown to facilitate p62-dependent autophagic degradation of STING by enhancing the interaction between p62 and STING, which contributes to immune homeostasis. Conversely, upon HSV-1 infection, SESN1 is downregulated to stabilize STING, leading to a potentiated cGAS-STING response that enhances antiviral defense [94].

Beyond STING itself, the downstream components TBK1 and IRF3 are also subjected to autophagic degradation as part of the negative feedback circuit. Multiple autophagy receptors are involved in the autophagic degradation of IRF3. The selective autophagy receptor NBR1 (neighbor of BRCA1 gene 1) has been found to bind IRF3 through its ubiquitin-associated (UBA) domain and promotes its degradation by autophagy [95]. Upon *Sendai virus* (SeV) infection, TRIM21 (tripartite motif containing 21) mediates K27-linked ubiquitination of IRF3, leading to its recognition by NDP52 and subsequent autophagic degradation [96]. The deubiquitinase OTUD7B (OTU deubiquitinase 7B) enhances p62 oligomerization, which functions as the IRF3-associated cargo receptor to facilitate its autophagic degradation, thereby preventing excessive immune responses [97]. In parallel, TBK1 can also be targeted for autophagic degradation by p62. It has been shown that SARS-CoV-2 helicase nonstructural protein 13 (NSP13) recruits TBK1 to p62 for autophagic degradation, enabling it to evade host immunity [98]. Similarly, other viruses have evolved strategies to subvert the cGAS-STING pathway via autophagy. For example, *Pseudorabies virus* (PRV) promotes the degradation of both cGAS and STING via autophagy by suppressing the mTORC1 complex [99]. These findings together suggest that autophagy receptors selectively remove STING, TBK1, and IRF3 rather than shutting down the pathway as a whole, and this substrate-specific degradation can either limit prolonged inflammation or be exploited by viruses to weaken antiviral defense.

Finally, the cGAS-STING pathway can also activate autophagy through a receptor-independent feedback mechanism. Small cytosolic DNA (scDNA) species (~20–40 bp) compete with long dsDNA for cGAS binding, which in turn promotes the association of cGAS with Rubicon [69,100]. This interaction enhances PI3KC3 activity and consequently triggers autophagic degradation of both STING and long dsDNA [100].

## Mitophagy in mtDNA-mediated cGAS-STING signaling

### Mechanisms of mtDNA release and cGAS-STING activation

**Figure 4.**
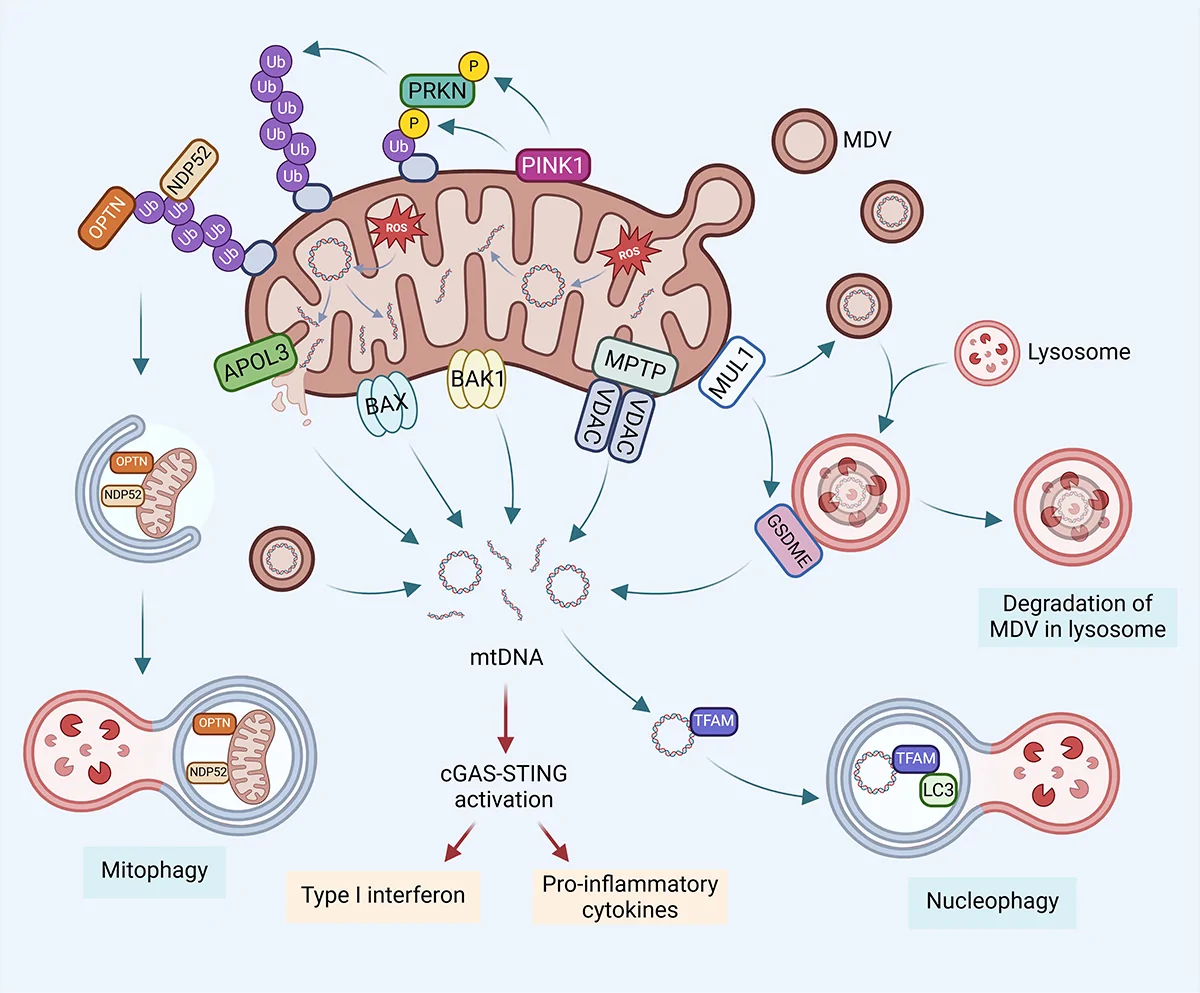
Mechanisms in regulation of the mtDNA-cGAS-STING axis. Under oxidative stress, reactive oxygen species (ROS) promote the cleavage of mtDNA into smaller double‑stranded mtDNA fragments. The fragmented mtDNA could be released into the cytosol via BAX-BAK1 oligomerization and mPTP-VDAC-mediated permeabilization. In addition, MUL1 promotes the package of mtDNA into MDVs and their trafficking to lysosomes. On the other hand, MUL1 could also promote caspase-dependent GSDME activation to trigger pore formation on the lysosome membrane, which facilitates mtDNA release from degraded MDVs within the lysosome. Moreover, lysosomal damage can also induce sublethal BAX-BAK1-mediated MOMP, allowing APOL3 to access and permeabilize the cardiolipin-rich inner mitochondrial membrane and thereby promote mtDNA release into the cytosol. The released mtDNA acts as cytosolic DNA to activate the cGAS-STING pathway, driving expression of type I interferons and pro-inflammatory cytokines. To limit excessive inflammatory activation, cells employ various autophagic mechanisms to control mtDNA availability: (1) PINK1-PRKN-mediated mitophagy: upon mitochondrial damage, PINK1 accumulates on the outer mitochondrial membrane, where it phosphorylates ubiquitin and PRKN, which promotes mitochondrial ubiquitination and the recruitment of autophagy receptors OPTN and NDP52 to target damaged mitochondria for autophagic degradation. (2) Nucleophagic clearance of cytosolic mtDNA: free mtDNA bound to TFAM is targeted for autophagic degradation via direct interaction of TFAM with LC3 via its LIR motif, resulting in its sequestration into autophagosomes for lysosomal clearance. (3) lysosomal degradation of MDVs: MDVs containing mtDNA are delivered to lysosomes for degradation, thus restricting mtDNA access to the cytosol. The diagram is generated using BioRender (https://BioRender.com).

Mitochondrial DNA (mtDNA) is a potent DAMP, when released into the cytosol, engages the DNA sensor cGAS and activates the STING pathway, driving type I interferon responses and inflammation [101]. Several distinct mechanisms govern mtDNA egress from mitochondria under stress (Figure 4). The classical pathway involves mitochondrial outer membrane permeabilization (MOMP) mediated by the pro-apoptotic proteins BAX (proteins BCL2 associated X) and BAK1 (BCL2 antagonist/killer 1). Upon activation, BAX and BAK1 oligomerize to form macropores in the outer membrane, allowing the release of mtDNA into the cytosol [102]. Additionally, the mitochondrial permeability transition pore (mPTP), a nonselective channel that opens in the inner membrane under conditions of calcium overload or oxidative stress, has been shown to facilitate mtDNA release. mPTP opening often works in concert with VDAC (voltage-dependent anion channel) oligomerization in the outer membrane to mediate mtDNA leakage under oxidative stress [103]. Oxidation of mtDNA and its fragmentation is a critical upstream event to enable its release. Reactive oxygen species (ROS) generated during mitochondrial stress mediates the oxidation of guanine bases to 8-oxo-7,8-dihydroguanine (8-oxoG) in mtDNA, forming oxidized mitochondrial DNA (ox-mtDNA). The structure-specific endonuclease FEN1 (flap structure-specific endonuclease 1) is activated by oxidative stress and cleaves ox-mtDNA into smaller double-stranded fragments within the mitochondrial matrix. Once fragmented, these ox-mtDNA fragments were released via mPTP-VDAC to activate the cGAS-STING pathway [104]. It has been reported that oxidized DNA is resistant to TREX1-mediated degradation and therefore remains available for cGAS-STING activation [105]. Therefore, the oxidation of mtDNA may be important not only for FEN1-dependent fragmentation and release but also for immune persistence.

To prevent persistent inflammation caused by cytoplasmic mtDNA, cells have evolved additional quality control mechanisms that operate downstream of mtDNA release (Figure 4). One such mechanism is nucleoid-phagy, a recently identified selective autophagic process that clears mislocalized mtDNA from the cytoplasm. Under stress conditions, TFAM (transcription factor A, mitochondrial), a key protein involved in mtDNA packaging and organization, accompanies mtDNA into the cytoplasm and acts as an autophagy receptor by directly interacting with LC3B, thereby delivering cytoplasmic mtDNA to autophagosomes for lysosomal degradation [106,107]. This additional layer of quality control may help limit excessive mtDNA-mediated immune activation while preserving responses to mitochondrial stress.

### Mitophagy as a negative regulator of mtDNA-driven cGAS-STING signaling

Mitophagy is a selective form of autophagy that eliminates damaged or superfluous mitochondria, thereby maintaining quality and quantity of mitochondria. The core mechanisms of mitophagy can be broadly classified into two pathways: the PINK1 (PTEN-induced kinase 1) /PRKN-mediated pathway and the receptor-mediated pathway involving mitophagy receptors such as BNIP3L/NIX (BCL2 interacting protein 3 like) and BNIP3 (BCL2 interacting protein 3). Both pathways converge on the autophagic machinery to degrade mitochondria [108]. The ubiquitin-dependent mitophagy pathway is governed by the PINK1-PRKN pathway (Figure 4). Under basal conditions, PINK1 is rapidly imported into healthy mitochondria and degraded. Upon mitochondrial depolarization or damage, PINK1 accumulates on the outer mitochondrial membrane, where it recruits and activates the E3 ubiquitin ligase PRKN to ubiquitinate outer membrane proteins. Ubiquitinated mitochondria are subsequently recognized by autophagy receptors such as OPTN (optineurin), and NDP52, leading to the clearance of damaged mitochondria via the autophagosome-lysosome pathway (for detailed mechanisms, see [109,110]). Receptor-mediated mitophagy operates independently of ubiquitination and relies on mitophagy receptors such as FUNDC1 (FUN14 domain containing 1), BNIP3L and BNIP3. These outer membrane proteins contain an LC3-interacting region (LIR) that allows them to directly bind LC3 on nascent autophagosomes, thereby tethering mitochondria to the forming autophagosome (for detailed mechanisms, see [111]).

By removing damaged mitochondria, mitophagy can reduce the availability of mtDNA capable of activating the cGAS-STING pathway. In aging brain, it has been revealed that cytosolic DNA released from perturbed mitochondria elicits cGAS activity in old microglia, leading to activation of STING, which in turn triggers reactive microglial transcriptional states, neurodegeneration, and cognitive decline [112]. This study positions the mtDNA-cGAS-STING pathway as an important contributor to age-associated neuroinflammation and neurological decline. Importantly, pharmacological induction of PINK1-PRKN-mediated mitophagy with urolithin A in old mice has been shown to reduce cytosolic mtDNA, attenuate cGAS/STING activation and ameliorate age-associated neurological decline [113]. Moreover, NAD+ supplementation reinforces PINK1-PRKN-dependent mitophagy to inhibit the cGAS-STING signaling, thereby attenuating neuroinflammation and cellular senescence in the brains of AD mice [114]. Together, these studies support PINK1-PRKN-dependent mitophagy enhancement as a potential strategy for limiting mtDNA-dependent cGAS-STING activation and neuroinflammation.

### OPTN in the interplay between mitophagy and STING signaling

OPTN is a multifunctional autophagy receptor involved in mitophagy, TBK1 activation, and Golgi membrane dynamics, raising the possibility that it connects mitophagy with STING signaling. During PINK1-PRKN-mediated mitophagy, OPTN is phosphorylated by TBK1, which enhances its recruitment to damaged mitochondria [115,116]. OPTN in turn forms a positive feedback loop to activate TBK1, thereby accelerating the mitophagy process [117]. As TBK1 is an essential kinase in the STING signaling, these findings raised the possibility that OPTN might also influence this pathway. Indeed, multiple studies have reported the localization of OPTN at the Golgi apparatus under different physiological or pathological conditions [118,119]. Moreover, OPTN plays an important role in Golgi homeostasis. In mammalian cells, OPTN was identified as a MYO6 (myosin VI)-binding partner that maintains Golgi architecture and its depletion causes Golgi fragmentation and defective exocytosis [120]. Amyotrophic lateral sclerosis (ALS)-linked OPTN mutations similarly disrupt Golgi structure and induce ER stress [121]. Moreover, TBK1 phosphorylates OPTN at Ser177, leading to ectopic OPTN accumulation and fragmentation of the Golgi apparatus, suggesting a feedback loop linking TBK1-OPTN axis to Golgi dynamics [122]. OPTN also participates in Golgi membrane-associated degradation (GOMED), a process morphologically similar to autophagy, by binding K33-polyubiquitinated substrates and facilitating their clearance [123]. These findings together reinforce the importance of OPTN in Golgi dynamics and functions.

Given the established role of OPTN in Golgi organization and the importance of the Golgi as a platform for STING-TBK1 signaling, it is plausible that OPTN modulates STING activity by regulating Golgi architecture and function. Emerging evidence suggests a functional connection between OPTN and STING signaling. First, it has been shown that the N-terminal domain of STING directly binds to the C-terminal ubiquitin-binding domain of OPTN, and knockdown of OPTN enhances the STING signaling [124]. In line with this, OPTN is recruited to STING-induced ubiquitin- and LC3B-labeled perinuclear vesicles in a mechanism depending on TBK1, which is involved in signaling downstream of interferon β [125]. More recently, we showed that STING promotes PINK1-PRKN-mediated mitophagy by activating TBK1 and enhancing OPTN recruitment to damaged mitochondria [126,127]. These findings support a role for OPTN as a functional link between STING signaling and mitophagy. Whether the Golgi-associated functions of OPTN, including Golgi fragmentation and GOMED, also contribute to STING signal attenuation remains to be determined.

### The role of lysosomes in regulation of the cGAS-STING pathway

#### Lysosomal DNA and cGAS-STING activation

Lysosomes have long been recognized as the terminal degradative compartment where key components of the cGAS-STING pathway are eventually degraded to terminate immune signaling. However, emerging evidence has revealed that lysosomes are not only passive endpoints of degradation but can also contribute to innate immune activation when DNA degradation fails or mtDNA is released from lysosomal compartments. Under normal conditions, lysosomal DNase II efficiently degrades internalized self-DNA. When this capacity is compromised, self-DNA accumulates in lysosomes and is subsequently sensed by cGAS, leading to constitutive STING activation and sustained type I interferon production [128]. In multiple LSDs models, lysosomal dysfunction similarly causes pathological accumulation of cytosolic dsDNA and cGAS proteins near lysosomes, which results in activation of the cGAS-STING signaling, leading to neuronal death, neuroinflammation and motor dysfunction [129].

Beyond storing DNA, lysosomes also receive mtDNA via mitochondrial-derived vesicles (MDVs) [130,131]. A recent study showed that MUL1/MAPL (mitochondrial E3 ubiquitin protein ligase 1), an outer mitochondrial membrane SUMO E3 ligase, promotes the packaging of mtDNA into MDVs and their trafficking to lysosomes [132]. The same study further found that MUL1 activates caspases-3/7 that cleave GSDME (gasdermin E), and cleaved GSDME forms pores on the lysosomal membrane in a manner dependent on LRRK2, leading to mtDNA release from the lysosome, which subsequently activates both cGAS-STING and inflammasome-driven GSDMD (gasdermin D) cleavage, the final step essential for pyroptosis [132]. These findings suggest that lysosomes can exert opposing effects on cGAS-STING signaling. Efficient lysosomal DNA degradation limits innate immune activation, whereas impaired DNA clearance or lysosomal membrane permeabilization can convert lysosomes into a source of immunostimulatory DNA, including self-DNA and mitochondria-derived DNA. Thus, lysosomal integrity and degradative capacity may determine whether DNA-containing cargo is safely eliminated or instead converted into an innate immune stimulus.

### Emerging links between STING and LRRK2 at lysosomes

Lysosomes provide a platform on which STING signaling can influence LRRK2 activity, while LRRK2 status may in turn affect cGAS-STING responses. LRRK2 is dynamically recruited to damaged lysosomes, where it contributes to cellular responses that preserve lysosomal integrity [133]. STING-mediated GABARAP lipidation has been shown to recruit and activate LRRK2 at the lysosomal surface [134]. The most common pathogenic variant in LRRK2 is the G2019S gain-of-function mutation, which increases LRRK2 kinase activity and is the leading genetic cause of familial PD [135]. This G2019S mutation is also associated with mtDNA damage and pharmacological suppression of the G2019S selective LRRK2 kinase activity abrogates mitochondrial DNA damage [136]. In line with this, this G2019S mutation has been linked to mitochondrial dysfunction and mtDNA leakage [137]. Although there is no direct evidence linking LRRK2 G2019S mutation to mtDNA-driven cGAS-STING activation, a recent study reveals that loss of LRRK2 in macrophages and microglia suppresses cGAS-STING signaling [138]. Strikingly, deficiency of VPS13C (vacuolar protein sorting 13 homolog C), a lipid transfer protein localized to contact sites between the ER and late endosomes/lysosomes and is linked to the genetic cause of early-onset PD, leads to accumulation of the lipid di-docosahexaenoyl bis (monoacylglycerol) phosphate (di-22:6-BMP) in lysosomes, a biomarker of the PD-associated LRRK2 G2019S mutation [139]. This lysosomal lipid dysregulation triggers the STING signaling activation by causing leakage of mtDNA into the cytosol and defective lysosomal clearance of activated STING [139]. These observations raise the possibility that LRRK2 activity, VPS13C-dependent lysosomal lipid homeostasis, and mtDNA-STING signaling are functionally connected at lysosomes, with potential relevance to PD pathogenesis.

### APOL3-mediated mtDNA release following lysosomal damage

Mitochondria are believed to originate from an endosymbiotic event in which a bacterial ancestor was engulfed by a host cell, eventually evolving into the organelle essential for eukaryotic energy metabolism and signaling [140]. This evolutionary history is preserved in the unique lipid composition of the inner mitochondrial membrane (IMM), which contains cardiolipin, a phospholipid found exclusively in bacterial membranes and the IMM, serving as a molecular fossil of endosymbiosis [141]. The IMM therefore retains a bacterial-like lipid environment that can be targeted by effectors originally evolved to combat cytosolic bacteria.

In 2021, it was discovered that the antibacterial effector APOL3 (apolipoprotein L3) induced by IFN-γ uses a detergent-like mechanism to kill intracellular bacteria by dissolving the lipid bilayers of invading bacteria [142]. Building on this, the same group recently reported that APOL3 can repurpose this membrane-disrupting activity to link lysosomal damage with mtDNA release and STING signaling [143]. The pathway operates in two steps. First, transient lysosomal damage triggers sub-lethal MOMP via BAK1-BAX, exposing the IMM without permeabilizing it. Second, APOL3 targets mitochondria that have already undergone MOMP and selectively permeabilizes the IMM by solubilizing cardiolipin, analogous to its detergent-like antibacterial mechanism. This allows mtDNA efflux into the cytosol to activate cGAS-STING without inducing cell death. Unlike classic mtDNA release through BAK1-BAX or mPTP pores, this pathway exposes the bacterial-like IMM via sublethal MOMP, allowing the detergent-like effector APOL3 to selectively permeabilize it and unleash mtDNA for cGAS-STING activation. These findings suggest that exposure of the bacterial-like mitochondrial inner membrane may be sensed as a danger signal during lysosomal stress.

### The endolysosomal compartments serve as a platform for STING -NF-κB signaling

One of the key functions of the cGAS-STING pathway is to activate the NF-κB pathway. While the underlying mechanism remains elusive, a recent study reported that STING activates NF-κB in a delayed manner following its exit from the Golgi to endolysosomal compartments [144]. Mechanistically, monomeric IRF3 is recruited to STING pS358, with delayed kinetics relative to its recruitment to STING pS366 (which promotes type I IFN responses). IRF3 engagement with STING pS358 induces trafficking to late endolysosomal compartments, supporting recruitment of TRAF6 (TNF receptor associated factor 6) and activation of NF-κB [144]. This activation pattern was reported to be conserved across the tetrapod models examined and independent of type I IFN signaling, suggesting a spatially and temporally restricted mode of STING-dependent NF-κB activation during endolysosomal trafficking. It is tempting to speculate that the rapid type I interferon response promotes cell survival and antiviral defense, whereas the delayed NF-κB output may, under certain conditions, drive pathological inflammation or cell death. Whether this temporal and spatial segregation of two inflammatory signals reflects a trade off between protective immunity and detrimental inflammation is an intriguing question for future investigation.

## Pathophysiological and therapeutic relevance

### Evidence from genetic models, disease models, and patient studies

Compared with the extensive mechanistic evidence obtained in cultured cells, the contribution of cGAS-STING-autophagy crosstalk to human disease remains less well defined. Nevertheless, emerging data from genetic models, disease models, and patient studies link dysregulation of these pathways to aging and neurodegeneration, acute and chronic organ injury, and autoimmune disease. The strength of the evidence varies considerably. Some studies use genetic epistasis to place defective autophagy or mitophagy upstream of STING-dependent pathology, whereas others primarily report associations among mitochondrial damage, circulating mtDNA, inflammatory markers, and disease severity.

Much of the current evidence in aging and neurodegeneration concerns defective mitophagy. In Parkin-mutant Drosophila, deletion of *Sting* ameliorated mitochondrial abnormalities, muscle degeneration, and locomotor impairment, indicating that STING contributes to the tissue damage caused by Parkin deficiency [145]. However, sting deletion produced a weaker rescue in pink1-mutant flies, suggesting that the pathological contribution of STING is not identical across different defects in the PINK1-PRKN pathway. In the aging mice brain, cytosolic DNA from perturbed mitochondria activates cGAS-STING in microglia, leading to STING-dependent neurodegeneration and cognitive decline [112]. Moreover, mitophagy has been shown to curtail cytosolic mtDNA-driven cGAS/STING activation by removing damaged mitochondria and limiting mtDNA leakage [113]. Notably, mitophagy was not uniformly reduced across aged tissues in this study. The relevant defect may therefore be an imbalance between the increasing burden of mitochondrial damage and the capacity of mitophagy to remove it, rather than a general decline in mitophagy itself. In line with this, enhancing mitophagy with urolithin A reduced cytosolic mtDNA accumulation, cGAS-STING activation and improved neurological function in aged mice [113]. Clinical observations provide additional support for mitochondrial damage-associated inflammation. Patients with PRKN- or PINK1-associated parkinsonism showed increased circulating cell-free mtDNA and IL-6 [146]. However, cGAS-STING activity was not directly assessed in patient tissues.

Mitochondrial damage-dependent activation of cGAS-STING has also been implicated in kidney diseases, cardiovascular diseases and autoimmune diseases. In cisplatin-induced acute kidney injury, mitochondrial injury in renal tubular cells was accompanied by cytosolic mtDNA accumulation and cGAS-STING activation, whereas Sting deficiency reduced renal inflammation and tissue injury [147]. Pink1 deficiency in mice aggravated cardiac hypertrophy by promoting cytosolic mtDNA release and subsequent activation of cGAS-STING signaling. Conversely, cardiomyocyte-specific PINK1 overexpression showed protective effects, while deletion of Sting partially reversed the cardiac abnormalities caused by Pink1 deficiency [148]. Several studies of autoimmune disease also support the close association of impaired mitophagy and mtDNA-triggered inflammation. IRGM1 (immunity-related GTPase M) deficiency in mice has been shown to develop defective mitophagy, resulting in mtDNA release, type I interferon responses, and multiorgan autoimmunity [149]. A more recent study reports that PINK1-Parkin-mediated mitophagy prevents mtDNA release to suppress cGAS-STING activation in a mouse model of autoimmune thyroiditis [150]. These findings therefore place STING downstream of impaired PINK1-mediated mitophagy and show how defective mitochondrial clearance can be converted into sustained inflammation, tissue remodeling, and the ultimate organ dysfunction.

Although current evidence remains largely derived from experimental models, and the extent to which mitophagy controls cGAS-STING activity in human disease requires further validation, these findings suggest cytosolic mtDNA as a recurrent driver of cGAS-STING activation across diverse disease settings and place mitophagy as an important mechanism that limits this inflammatory signal. Enhancing mitophagy may therefore represent a promising strategy for diseases in which persistent mtDNA accumulation contributes to chronic inflammation.

### Therapeutic potential and challenges

The growing involvement of cGAS-STING signaling in inflammatory, autoimmune, degenerative, and malignant diseases has stimulated considerable interest in pharmacological modulation of this pathway. Both cGAS and STING agonists and inhibitors have been developed (see [151,152]). In diseases characterized by persistent sensing of endogenous DNA and chronic inflammation, inhibition of cGAS-STING represents a rational therapeutic strategy. Conversely, activation of STING can enhance innate and adaptive antitumor immunity and has therefore been extensively explored in cancer therapy [153]. The opposing therapeutic strategies highlight an important feature of this pathway that cGAS-STING activity is not intrinsically protective or pathogenic, and the desired direction of intervention depends on the source and duration of DNA sensing and the disease context.

The close relationship between autophagy and cGAS-STING provides an additional level at which this pathway could be therapeutically modified. In diseases driven by mitochondrial damage and persistent mtDNA release, enhancing mitochondrial quality control may reduce the inflammatory stimulus upstream of cGAS rather than directly inhibiting immune signaling. For example, induction of mitophagy by urolithin A reduced cytosolic mtDNA accumulation and cGAS-STING activation in aged mice, whereas restoration of PINK1-dependent mitophagy protected against pathological cardiac hypertrophy associated with mtDNA-STING signaling [113,148]. By contrast, inhibition of autophagic degradation could theoretically prolong STING signaling when stronger immune activation is desirable, particularly in cancer [153]. However, inhibiting autophagy to prolong STING signaling may also increase endogenous DNA accumulation through defective organelle clearance, thereby affecting the pathway at both upstream and downstream levels. These considerations argue against broadly activating or inhibiting autophagy as a uniform strategy for manipulating cGAS-STING. Therapeutic responses are likely to depend on which autophagic process is altered and which step of the cGAS-STING pathway is affected. Enhancing mitophagy or DNA clearance may be beneficial when endogenous DNA accumulation drives chronic inflammation, whereas preventing the degradation of STING or its downstream effectors could increase immune activation in settings such as cancer. An additional challenge is that STING itself induces non-canonical autophagy and lysosomal responses that are mechanistically distinct from canonical macroautophagy. Future therapeutic strategies should therefore distinguish between targeting canonical autophagic clearance and STING-induced non-canonical autophagy, rather than treating autophagy as a single pathway.

## Conclusions and perspectives

The cGAS-STING pathway and autophagy form a complex, bidirectional regulatory network that operates at multiple subcellular levels. A major conceptual advance is the recognition that STING possesses an evolutionarily ancient, TBK1-independent capacity to induce noncanonical autophagy. More recent studies further link this STING function to TFEB-dependent lysosome biogenesis and lysosomal quality control, although the physiological breadth of these functions remains to be established. The autophagic activity of STING appears to predate its canonical immune signaling and broadens its role beyond interferon induction to the regulation of membrane dynamics. Conversely, autophagy pathways provide multiple negative-feedback mechanisms that restrain cGAS-STING activation through clearance of cytosolic DNA, selective degradation of pathway components (cGAS, STING, TBK1, IRF3), and organelle selective autophagy, including mitophagy, Golgiphagy, and ER-phagy as well as ESCRT-mediated microautophagy.

Several limitations should be considered when interpreting the current evidence. The form of autophagy examined is not always clearly defined, as changes in LC3 lipidation and p62 abundance are frequently used as the principal readouts without establishing the membrane compartment or degradative route involved. In addition, most proposed mechanisms have been established in cultured cells subjected to acute pharmacological, genetic, or nucleic acid stimulation. Evidence from genetic animal models, disease models, and patient samples remains limited and uneven, making it difficult to determine which mechanisms operate in vivo and contribute substantially to disease. Consequently, the therapeutic value of targeting autophagy or cGAS-STING, including whether activation or inhibition would be beneficial in a given disease, remains insufficiently established.

Several key questions remain unanswered. How do different membrane sources, including the ER-Golgi intermediate compartment, the Golgi, recycling endosomes, and lysosomes, coordinate the spatiotemporal activation of STING-driven noncanonical autophagy? What determines whether STING-induced LC3 lipidation proceeds on double membrane autophagosomes in a WIPI2-dependent manner or on single membrane vesicles in a V-ATPase-ATG16L1 dependent manner? Moreover, do the membrane origins of these two structures differ? In the reported STING-induced autophagy models, double membrane autophagosomes arise mainly from the ERGIC, while single membrane vesicles may derive from the Golgi or recycling endosomes. Do their lipid transfer mechanisms also diverge? Does STING-induced membrane lipidation employ the ATG9-ATG2 complex for lipid transport? Addressing these questions should help clarify how distinct membrane sources support different STING-dependent autophagic outputs.

The precise molecular links between lysosomal damage, mtDNA release, and cGAS-STING activation via the APOL3 and MUL1-gasdermin pathways have only recently emerged. How these pathways integrate with mitophagy to prevent sterile inflammation requires further investigation. Although much attention has focused on the role of cGAS-STING in immune cells and inflammatory responses, its noncanonical autophagic functions may also operate in nonimmune cells to maintain broader cellular homeostasis. Exploring these potential physiological functions will help clarify whether the evolutionarily conserved autophagy function of STING serves primarily as an acute stress response or as a housekeeping mechanism that operates continuously under basal conditions.

Recent evidence indicates that STING can promote PINK1-PRKN-mediated mitophagy through TBK1-dependent recruitment of OPTN to damaged mitochondria, which provides a direct link between STING signaling and mitochondrial quality control. However, several mechanistic questions remain unresolved. It is currently unclear whether STING-dependent mitophagy relies exclusively on the canonical PINK1-PRKN machinery or also engages components of the STING-induced noncanonical autophagy pathways described above. Another important question is how STING recognizes damaged mitochondria. Mitochondrial injury alters membrane organization and can expose inner-membrane lipids such as cardiolipin. It is unclear whether and how these changes contribute to STING recruitment or activation at damaged mitochondria. The physiological and disease contexts in which STING-dependent mitophagy operates, and how this protective response is coordinated with the inflammatory outputs of STING signaling, also warrant further investigation. Moreover, whether STING participates in other forms of selective autophagy, such as pexophagy, lysophagy or ER-phagy, is worth investigating in the future. Addressing these questions should clarify how innate immune sensing intersects with selective autophagy and organelle quality control under cellular stress.

## Abbreviations

Abbreviation: 2’3’-cGAMP, 2’3’-cyclic GMP-AMP; 8-oxoG, 8-oxo-7,8-dihydroguanine; ADGRE5/CD97, adhesion G protein-coupled receptor E5; AGS, Aicardi-Goutières syndrome; AIM2, absent in melanoma 2; ALIX, ALG-2 interacting protein X; ALS, Amyotrophic lateral sclerosis; APOL3, apolipoprotein L3; ARF1, ARF GTPase 1; ATG, autophagy-related; ATP, adenosine triphosphate; ATP6V0C, ATPase H+ transporting V0 subunit c; BAK1, BCL2 antagonist/killer 1; BANF1/BAF, barrier to autointegration nuclear assembly factor 1; BAX, BCL2 associated X; BNIP3, BCL2 interacting protein 3; BNIP3L/NIX, BCL2 interacting protein 3 like; CALCOCO2/NDP52, calcium binding and coiled-coil domain 2; cGAS, cyclic GMP-AMP synthase; chaperone-mediated autophagy (CMA), chaperone-mediated autophagy; cryo-EM, cryo-electron microscopy; CRL5, cullin-RING ubiquitin ligase 5; DAMPs, damage-associated molecular patterns; di-22:6-BMP, di-docosahexaenoyl bis(monoacylglycerol) phosphate; dsDNA, double-stranded DNA; ER, endoplasmic reticulum; ERGIC, ER-Golgi intermediate compartment; ESCRT, endosomal sorting complexes required for transport; FEN1, flap structure-specific endonuclease 1; FIG4, FIG4 phosphoinositide 5-phosphatase; FLCN, folliculin; FNIP, folliculin interacting protein; FUNDC1, FUN14 domain containing 1; GABARAP, GABA type A receptor-associated protein; GABARAPL1, GABA type A receptor associated protein like 1; GABARAPL2, GABA type A receptor associated protein like 2; GAPs, GTPase-activating proteins; GATOR1, Gap Activity TOward Rags 1; GATOR2, GTPase activating proteins toward Rags subcomplex 2; GOMED, Golgi membrane-associated degradation; GSDMD, gasdermin D; GSDME, gasdermin E; GTP, guanosine triphosphate; HOPS, homotypic fusion and protein sorting; IFI16, interferon gamma inducible protein 16; IFNs, interferons; IκBα, NF-κB inhibitor alpha; IKK, inhibitor of NF-κB kinase; IKKα, inhibitor of NF-κB kinase subunit alpha; IKKβ, inhibitor of NF-κB kinase subunit beta; IKKε, inhibitor of NF-κB kinase subunit epsilon; IL1B, interleukin 1 beta; IRF3, interferon regulatory factor 3; ISGs, interferon-stimulated genes; LIR, LC3-interacting region; LLPS, liquid-liquid phase separation; LPS, lipopolysaccharide; LRRK2, leucine rich repeat kinase 2; LSDs, lysosomal storage disorders; MAP1LC3/LC3, microtubule associated protein 1 light chain 3; MDVs, mitochondrial-derived vesicles; MITF, melanocyte inducing transcription factor; MOMP, mitochondrial outer membrane permeabilization; mPTP, mitochondrial permeability transition pore; mtDNA, mitochondrial DNA; mTORC1, mechanistic target of rapamycin complex 1; MUL1, mitochondrial E3 ubiquitin protein ligase 1; MYO6, myosin VI; NBR1, neighbor of BRCA1 gene 1; NEMO/IKKγ, inhibitor of NF-κB kinase subunit gamma; NF-κB, nuclear factor kappa B; OPTN, optineurin; OTUD7B, OTU deubiquitinase 7B; PAMPs, pathogen-associated molecular patterns; PAS, phagophore assembly site; PD, Parkinson’s disease; PDCD6IP, programmed cell death 6 interacting protein; PE, phosphatidylethanolamine; PI3KC3-C1, class III phosphatidylinositol 3-kinase complex I; PI4P, phosphatidylinositol 4-phosphate; PIK3C3/VPS34, phosphatidylinositol 3-kinase catalytic subunit type 3; PIK3R4/VPS15, phosphoinositide-3-kinase regulatory subunit 4; PIKFYVE, phosphoinositide kinase, FYVE-type zinc finger containing; PINK1, PTEN induced kinase 1; PRKN, parkin RBR E3 ubiquitin protein ligase; PRRs, pattern recognition receptors; PtdIns(3,5)P2, phosphatidylinositol-3,5-bisphosphate; PtdIns(4,5)P2, phosphatidylinositol-4,5-bisphosphate; PtdIns3P, phosphatidylinositol-3-phosphate; PtdInsPs, phosphatidylinositol phosphates; RAG, Ras related GTP binding; RB1CC1/FIP200, RB1 inducible coiled-coil 1; REs, recycling endosomes; RETREG1/FAM134B, reticulophagy regulator 1; RHEB, Ras homolog, mTORC1 binding; ROS, reactive oxygen species; RUBCN/Rubicon, rubicon autophagy regulator; scDNA, small cytosolic DNA; SENP2, SUMO specific peptidase 2; SENP3, SUMO specific peptidase 3; SESN1, sestrin 1; SLE, systemic lupus erythematosus; SNAP29, synaptosomal-associated protein 29; SNARE, soluble N-ethylmaleimide-sensitive factor attachment protein receptor; SPSB3, splA/ryanodine receptor domain and SOCS box containing 3; SQSTM1/p62, sequestosome 1; STING, stimulator of interferon genes; STX17, syntaxin 17; TAX1BP1, Tax1 binding protein 1; TBK1, TANK-binding kinase 1; TFAM, transcription factor A, mitochondrial; TFEB, transcription factor EB; TFE3, transcription factor binding to IGHM enhancer 3; TGN, trans-Golgi network; TLR4, toll like receptor 4; TLR9, toll like receptor 9; TRAF6, TNF receptor associated factor 6; TREX1, three prime repair exonuclease 1; TRIM21, tripartite motif containing 21; TRIM23, tripartite motif containing 23; TRIM38, tripartite motif containing 38; UBE2N, ubiquitin conjugating enzyme E2 N; ULK1, unc-51 like autophagy activating kinase 1; VAC14, VAC14 component of PIKFYVE complex; VAMP, vesicle-associated membrane protein; VAPs, VAMP-associated proteins; V-ATPase, vacuolar ATPase; VDAC, voltage-dependent anion channel; VPS13C, vacuolar protein sorting 13 homolog C; WIPI, WD-repeat protein interacting with phosphoinositides; WIPI2, WD repeat domain, phosphoinositide interacting 2; YKT6, YKT6 vesicular SNARE protein; ZBP1/DAI, Z-DNA binding protein 1.

## Data Availability

Data sharing is not applicable to this article as no data were created or analyzed.
